# Endogenous Isoquinoline Alkaloids Agonists of Acid-Sensing Ion Channel Type 3

**DOI:** 10.3389/fnmol.2017.00282

**Published:** 2017-09-13

**Authors:** Dmitry I. Osmakov, Sergey G. Koshelev, Yaroslav A. Andreev, Sergey A. Kozlov

**Affiliations:** ^1^Shemyakin-Ovchinnikov Institute of Bioorganic Chemistry, Russian Academy of Sciences Moscow, Russia; ^2^Institute of Molecular Medicine, Sechenov First Moscow State Medical University Moscow, Russia

**Keywords:** acid sensing ion channel (ASIC), signaling, neuroscience, oocyte, endogenous opioid

## Abstract

Acid-sensing ion channels (ASICs) ASIC3 expressed mainly in peripheral sensory neurons play an important role in pain perception and inflammation development. In response to acidic stimuli, they can generate a unique biphasic current. At physiological pH 7.4, human ASIC3 isoform (hASIC3) is desensitized and able to generate only a sustained current. We found endogenous isoquinoline alkaloids (EIAs), which restore hASIC3 from desensitization and recover the transient component of the current. Similarly, rat ASIC3 isoform (rASIC3) can also be restored from desensitization (at pH < 7.0) by EIAs with the same potency. At physiological pH and above, EIAs at high concentrations were able to effectively activate hASIC3 and rASIC3. Thus, we found first endogenous agonists of ASIC3 channels that could both activate and prevent or reverse desensitization of the channel. The decrease of EIA levels could be suggested as a novel therapeutic strategy for treatment of pain and inflammation.

## Introduction

Acid-sensing ion channels (ASICs) are voltage-independent ligand-gated cation channels related to the superfamily of amiloride-sensitive degenerin/epithelial Na^+^ channels (Kellenberger et al., 2002). In mammals, four genes encode six different subunits, four of which assemble into functional homomeric channels ASIC1a, ASIC1b, ASIC2a and ASIC3 (Wemmie et al., 2006). ASICs are localized on the postsynaptic membrane and can be activated by a rapid pH drop below 6.0, which in turn can lead to a membrane depolarization and trigger bursts of action potential (Mamet et al., 2002). ASIC3 channels expressed mainly in peripheral sensory neurons (Waldmann et al., 1997; Poirot et al., 2006) are of particular interest due to their biophysical and physiological properties. ASIC3 channels are able to generate a biphasic current containing a transient component followed by a non-desensitizing sustained current in response to acidic stimuli (Osmakov et al., 2014) and can integrate different inflammatory or ischemic stimuli (Immke and McCleskey, 2001; Allen and Attwell, 2002; Deval et al., 2008). Among such stimuli are compounds such as arachidonic acid, which, at a 1–10 μM range, increases the amplitude of both the transient and sustained components of the acid sensing ion channel (ASIC) current (Smith et al., 2007); serotonin, which increases the ASIC3-sustained current (Wang et al., 2013); and FMRFamide-related neuropeptides, potentiating the proton-gated current (Askwith et al., 2000). Some neuropeptides affecting opioid receptors (dynorphins) were also reported to potentiate ASIC channels (Sherwood and Askwith, 2009). Polyamine agmatine, as well as lysophosphatidylcholine and arachidonic acid, was shown to evoke a constitutive depolarizing ASIC3 current at resting physiological pH 7.4 at millimolar and micromolar concentrations, respectively (Li et al., 2010; Marra et al., 2016).

For a long time, endogenous isoquinoline alkaloids (EIAs) were considered biological active molecules although their function was not completely understood. Representatives of this group of compounds, such as tetrahydropapaveroline (THP) and reticuline, are precursors of endogenous morphine biosynthesis in mammals (Weitz et al., 1987). High circulating levels of THP (also known as norlaudanosoline) are associated with pathological states such as Parkinson’s disease and alcoholism (Sango et al., 2000; McCoy et al., 2003). It was shown that THP inhibits mitochondrial respiration and increases reactive oxygen species production (Surh and Kim, 2010), decreases dopamine biosynthesis by inhibiting tyrosine hydroxylase (Kim et al., 2005; Yao et al., 2010; Nowicki et al., 2015), and inhibits dopamine uptake by acting on its plasma membrane transporter (Okada et al., 1998). Reticuline, which differs from THP by three methyl groups, was detected in rat brains (Zhu et al., 2003), in the animal’s neural tissue (Zhu et al., 2002), and in cultured human cells (Poeaknapo et al., 2004). Reticuline demonstrated anti-platelet aggregation activity (Chen et al., 2000), showed butyrylcholinesterase inhibitory activity (Hošt’álková et al., 2015), and elicited peripheral vasodilation via reduction of the voltage-activated peak amplitude of L-type Ca^2+^-channel (Dias et al., 2004; Medeiros et al., 2009). Examination of the reticuline ability to mimic opioids exhibited low binding affinity to μ-opioid receptors in living cells (Zhu et al., 2004; Nikolaev et al., 2007). As a result, the role of THP and reticuline as neuronal receptor ligands has not been discovered to date.

ASIC3 channels significantly contribute to the perception and development of pain conditions, acid-mediated and inflammatory pain (Deval et al., 2008; Yen et al., 2009), and development of primary and/or secondary mechanical hypersensitivity in muscles (Sluka et al., 2009); participate in the perception of pain from mechanical stimuli (Jones et al., 2005); and are involved in the perception of pain signals from the lungs and gastrointestinal tract (Wultsch et al., 2008). However, it is unknown if the acidification (proton concentration rise) or release of unidentified endogenous ligands should be considered as a pathological impact (Krishtal, 2015). Here, we demonstrated the ability of two endogenous molecules—THP and reticuline (PubChem CID 18519 and 10233, respectively) to activate human and rat ASIC3 (rASIC3) channels at physiological pH, as well as prevent a steady-state desensitization of the channels.

## EIAs Self-Sufficiently Activate Human and Rat ASIC3

**Figure 1:**
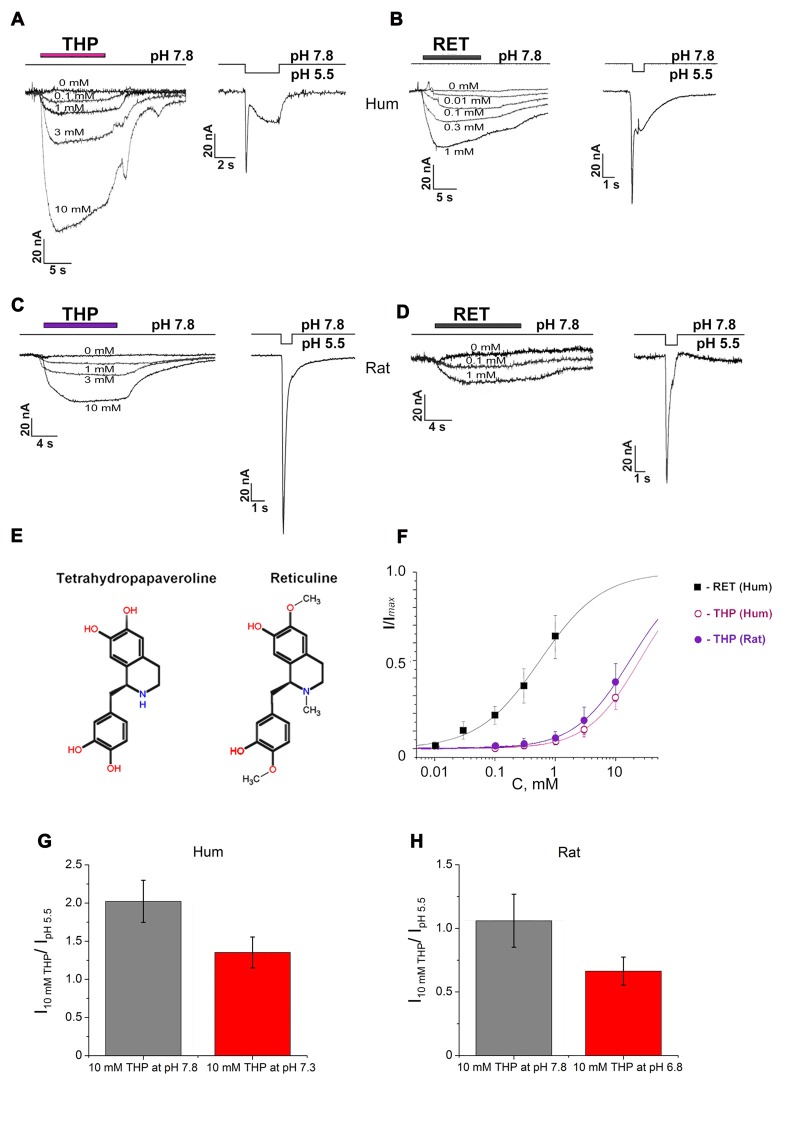
Activating effect of endogenous isoquinoline alkaloids (EIAs) on Acid-sensing ion channels (ASIC3) channels. Action of tetrahydropapaveroline (THP; **A,C**) and reticuline **(B,D)** shown together with response to control stimulus for human **(A,B)** and rat **(C,D)** channels expressed in oocytes using whole cell configuration. Currents were measured at a holding pH 7.8 and were compared to control currents (H^+^ induced response by pH drop to 5.5) on the same cell. **(E)** Chemical structure of THP and reticuline. **(F)** Dose-response curves for the EIAs’ activation effect on human and rat ASIC3 (rASIC3) channels. Data were fitted by the logistic equation. *I*_max_ is a maximal amplitude predicted by the logistic equation fitting of EIAs’ induced currents data. **(G)** Activation effect of THP (10 mM) on human ASIC3 (hASIC3) channels at conditioning pH 7.8 (gray column) and 7.3 (red column). **(H)** Activation effect of THP (10 mM) on rASIC3 channels at conditioning pH 7.8 (gray column) and 6.8 (red column). Each point is presented as mean ± SE of 4–5 measurements.

The ability of EIAs to activate ASIC3 channels was studied in whole-cell configuration on oocytes of *Xenopus laevis*. THP and reticuline caused slowly activated sustained inward currents both in human (Figures 1A,B) and rat (Figures 1C,D) homomeric ASIC3 channels at resting pH 7.4 and above.

THP (Figure 1E) dose-dependently activated the channels at pH 7.8, and the activation did not reach a maximal value at the highest accessible concentration (10 mM). In the case of human ASIC3 channels (hASIC3), the activating effect was greater and even exceeded the response of the cell to pH drop of 7.8–5.5 in control experiments (Figure 1A). The maximal effect reached about 30% of *I*_max_ (a maximal amplitude predicted by the logistic equation fitting of dose-dependence of EIA-induced current). According to steady fitting by a logistic equation, the half-maximal effective concentration (EC_50_) and the Hill coefficient (*n*_H_) for THP were 24.86 ± 1.61 mM and 0.99 ± 0.05, respectively (*n* = 5; Figure 1F). THP shown the same potency on the rASIC3 channel (rASIC3; Figure 1C). The activating effect under the same conditions reached about 30% of *I*_max_. The calculated EC_50_ and *n*_H_ values were 17.23 ± 0.75 mM and 0.95 ± 0.04, respectively (*n* = 5; Figure 1F).

Reticuline (Figure 1E) is a hydrophobic substance that does not have a high solubility in physiological solutions for electrophysiology. Consequently, a maximal concentration of 1 mM was used in experimentations. The maximal effect on hASIC3 reached about 60% of *I*_max_. Steady fitting by a logistic equation gave EC_50_ and *n*_H_ values of 0.56 ± 0.04 mM and 0.85 ± 0.05, respectively (*n* = 5; Figure 1F).

**Figure 2:**
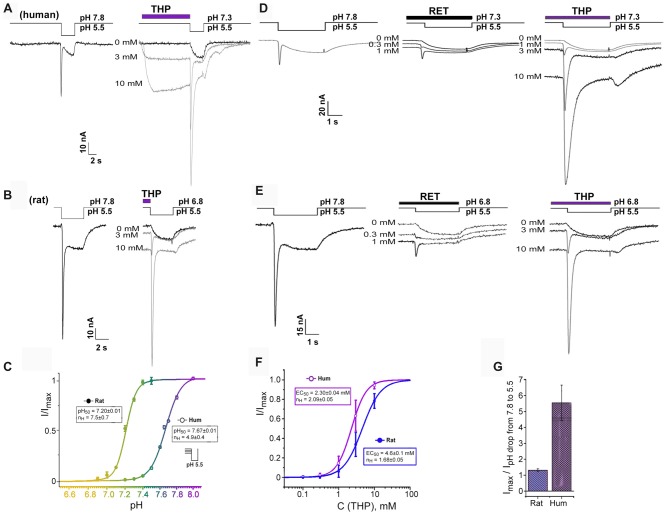
Transient current recovering effect of EIAs on ASIC3 channels. **(A,B)** Dual effect of THP preincubation reflected in sustained current generation together with inhibition of steady-state desensitization. Whole-cell currents recorded from human **(A)** or rat **(B)** ASIC3 channels held at pH 7.3 and 6.8, respectively were measured with pH 5.5 stimulus at different THP concentrations. **(C)** ASIC3 steady-state desensitization of human and rat channels by increasing proton concentrations in the conditioning period. Each point is presented as mean ± SE of seven measurements. **(D,E)** Traces for human **(D)** and rat **(E)** ASIC3 currents measured for non-desensitized channel state at resting pH 7.8 (left) and for desensitized channel state in the presence of reticuline (in the center) and THP (on the right). **(F)** Dose-response curve for THPs’ transient current recovering effect on hASIC3 and rASIC3. *I*_max_ was predicted by the fitting of dose dependences by a logistic equation independently for each cell. Each point is presented as mean ± SE of 4–5 measurements. **(G)** Comparative chart for calculated maximal amplitude of transient currents (recovered by THP from desensitization) to measured transient currents in the control by a pH drop of 7.8–5.5. Data for rASIC3 (*n* = 4) and hASIC3 (*n* = 5) are presented as mean ± SE.

The same activation effect was produced by ligands when the channels were in the steady- state desensitization (Figures 2A,B). The decrease of conditioning pH produced reduction in the ligand effect both on human and rASIC3 channels (Figures 1G,H). Thus, an increase of proton concentration in conditioning solution attenuated a channel sensitivity to THP.

## Pharmacological Difference of hASIC3 and rASIC3

We found an important relationship of the rat and human ASIC3 channels’ responses to proton stimuli from different resting states. Response from bath pH 7.8–5.5 exhibited a “normal” biphasic current (i.e., with a transient component followed by a sustained one), whereas at physiological resting pH 7.3–7.4 (Street et al., 2001), hASIC3 responded to the pH drop to 5.5 by a sustained component of the current only while the transient component of the current was completely desensitized. In contrast, for rASIC3, resting pH 7.3–7.4 did not desensitize the transient component of the current. Therefore, rat and human ASIC3 channels need different pH values for transient component desensitization. We characterized this difference as the level of transient current amplitude evoked by pH 5.5 stimulation from variable resting pH for both channels (Figure 2C). The calculated pH_50_ of steady-state desensitization (the value of H^+^ concentration in an extracellular solution at which the transient current amplitude of response to pH stimulus is half-maximal) for hASIC3 was 7.67 ± 0.01 (*n*_H_ = 4.9 ± 0.4) and for rASIC3 was 7.20 ± 0.01 (*n*_H_ = 7.5 ± 0.7). Thus, the biphasic response of hASIC3 is almost impossible at normal physiological pH and below since channels are in the desensitized state. Limitations of the hASIC3 functioning in normal conditions appear to be abnormal. Therefore, we hypothesized the presence of an additional regulation mechanism in normal conditions. We checked if EIAs were able to change channel status from a desensitized to a closed state.

## EIAs Reverse Steady-State Desensitization

EIAs cause both the channel activation and transient component of hASIC3 current restoration from a desensitization at pH 7.3 (Figures 2A,D). For THP, the restoration effect was more pronounced. This effect was dose-dependent and reached a maximal possible value at the highest applied concentration of THP (10 mM). One major experimental problem was to choose an appropriate control for the dose dependence calculation since the transient current was completely desensitized at pH 7.3, and the transient current induced at a pH drop of 7.8–5.5 was significantly lower than one recovered by THP at a pH drop of 7.3–5.5. For this reason, the maximal amplitude of the transient current recovered by THP from desensitization (*I*_max_) was predicted by the fitting of the dose dependence by a logistic equation for each experimental cell. Finally, all data for THP were assembled together and fitted by a logistic equation with EC_50_ = 2.30 ± 0.04 mM and *n*_H_ = 2.09 ± 0.05 (*n* = 4–5, for each point; Figure 2F).

The same effect has been demonstrated on rASIC3 channels under mild acidic conditions (pH 6.8) when the transient component of rASIC3 current is desensitized completely (Figures 2B,E). Values of EC_50_ and *n*_H_ for THP were 4.6 ± 0.1 mM and 1.68 ± 0.05, respectively (*n* = 4–5; Figure 2F). Therefore, the THP reversed steady-state desensitization of rASIC3 was equally potent to that of hASIC3, but overall, the amplitude of the recovered transient current was significantly greater in the case of hASIC3 (when compared to the control transient current amplitude evoked by a pH drop of 7.8–5.5; Figure 2G).

## Perspectives for The Pain Treatment

We discovered the EIAs to be potent ligands of the ASIC3 channel. As it was shown earlier, EIAs’ blood levels increase under pathological conditions such as infection or inflammation (Glattard et al., 2010; Laux-Biehlmann et al., 2012). Increases in EIAs’ concentration may contribute to pain symptoms in people suffering from Parkinson’s disease, as evidenced by the correlation between reduced content of EIAs and pain relief (Laux-Biehlmann et al., 2013). In mammalian brain tissues, the EIAs concentration was estimated on 10–100 nanomolar level (Sango et al., 2000; Yao et al., 2010) and electron microscopy imaging detected these compounds only in presynaptic terminals in the cerebellum and postsynaptic terminals of the other brain regions (Laux et al., 2011). Therefore, we can expect that the concentration in synaptic cleft could reach hundreds of micromoles. However, molecular targets for these compounds were not identified.

The ability of EIAs to directly affect ASIC3 channels indicates that the functioning of acid-sensing channels is definitely controlled in the organism by ligands other than protons. EIA may produce positive regulation of nociception via ASICs opposite to analgesic action of other opioids via metabotropic opioid receptors (Cai et al., 2014). This possible bidirectional action could be an important goal for the nearest investigations. Rat and human ASIC3 channels shown a difference in responds to EIA application as well as these channels did not equally responded to acidification. This may be a reason for analgesic prodrug overestimating in preclinical trials.

**Figure 3:**
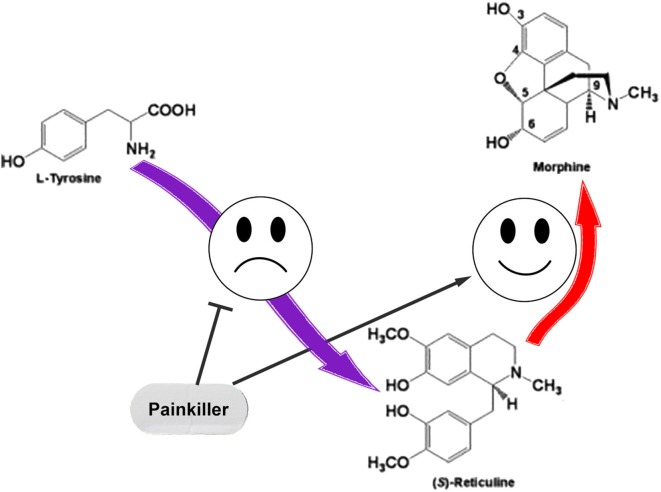
The simple representation for the endogenous pathway of morphine biosynthesis in mammals. Pain relief could be induced by inhibition of the reticuline/THP biosynthesis or by enhancement of their conversion to the morphine.

We assume that the regulation of ASIC sensitivity by endogenous non-proton ligands could be a part of the mutual regulation process of nociception and anti-nociception. The role for THP and (S)-reticuline as endogenous precursors of morphine in mammalian cells was reported (Poeaknapo et al., 2004) and cells most probably have a controlled system of alkaloid biosynthesis from tyrosine. Under certain conditions, neuronal cells can release pro-nociceptive and pro-inflammatory molecules (including THP and (S)-reticuline) causing initiation of pain and inflammation. To discontinue the process, the production of these molecules should be stopped. Additionally, THP and (S)-reticuline could be transformed to endogenous morphine that has the analgesic effect (Zhu et al., 2003). Consequently, the pathway of the endogenous morphine biosynthesis in mammals produces both pro- and anti- nociceptive molecules. Therefore, regulation of this pathway could be used as a target for therapeutic intervention. A decrease of EIA level by the inhibition of ways of their biosynthesis or/and activation of their further conversion could be proposed for the treatment of pain and inflammatory conditions (Figure 3).

## Materials and Methods

Chemical Reagents Reticuline and THP were obtained from Toronto Research Chemicals (Canada). Fresh solutions of reagents were made directly before testing.

### Electrophysiological Study on *Xenopus Laevis* Oocytes

Oocytes expressed hASIC3 and rASIC3 homomeric channels were prepared as described (Dubinnyi et al., 2012). After injection, the oocytes were kept for 2–3 days at 19°C and then up to 7 days at 15°C in an ND-96 medium containing (in mM): 96 NaCl, 2 KCl, 1.8 CaCl_2_, 1 MgCl_2_ and 10 HEPES titrated to pH 7.4 with NaOH supplemented with gentamycin (50 μg/ml). Two electrode voltage clamp recordings were made using a GeneClamp500 amplifier (Axon Instruments), and data were filtered at 20 Hz and digitized at 100 Hz by an AD converter L780 (L-Card, Moscow, Russia) using homemade software. A computer-controlled valve system for a fast solution switch was used. Microelectrodes were filled with 3 M KCl solutions. All solutions of the testing compounds were supplemented with 0.1% BSA. To induce currents, we employed ND-96-modified solutions in which 10 mM of HEPES was substituted for 5 mM MES pH 5.5. A set of external ND-96 solutions with pH 6.8–7.0 (buffered with 10 mM of MOPS), pH 7.1–8.0 (buffered with 10 mM of HEPES) was prepared.

### Data Analysis

The analysis of electrophysiological data was performed using the program OriginPro 8.6. The curve-fitting analysis was carried out with the following four-parameter logistic equation: *F*(*x*) = *A*/(1 + (*x*/*x*0)*n*), where *x* is the concentration of the ligand, *F*(*x*) is the response value at a given ligand concentration, *A* is the response value at maximal activation (% of control), x0 is the EC_50_ value and *n* is the Hill coefficient (slope factor). All data are presented as the mean ± SE.

## Author Contributions

DIO and SAK designed the experiment; DIO and SGK in assistance with YAA performed the experiments on oocytes; DIO, SGK, YAA and SAK analyzed the data and wrote the manuscript.

## Conflict of Interest Statement

The authors declare that the research was conducted in the absence of any commercial or financial relationships that could be construed as a potential conflict of interest.
